# Design of Novel Coumarin Derivatives as NUDT5 Antagonists That Act by Restricting ATP Synthesis in Breast Cancer Cells

**DOI:** 10.3390/molecules28010089

**Published:** 2022-12-22

**Authors:** Vidya Niranjan, Sanjana Jayaprasad, Akshay Uttarkar, Raviraj Kusanur, Jitendra Kumar

**Affiliations:** 1 Department of Biotechnology, R V College of Engineering, Bengaluru 560059, Karnataka, India; 2 Department of Chemistry, R V College of Engineering, Bengaluru 560059, Karnataka, India; 3 Bangalore Bioinnovation Centre (BBC), Helix Biotech Park, Electronics City Phase 1, Bengaluru 560100, Karnataka, India

**Keywords:** NUDT5, ATP synthesis, coumarin derivative, molecular dynamics simulation, metadynamics simulation, MTT assay

## Abstract

Breast cancer, a heterogeneous disease, is among the most frequently diagnosed diseases and is the second leading cause of death due to cancer among women after lung cancer. Phytoactives (plant-based derivatives) and their derivatives are safer than synthetic compounds in combating chemoresistance. In the current work, a template-based design of the coumarin derivative was designed to target the ADP-sugar pyrophosphatase protein. The novel coumarin derivative (2*R*)-2-((*S*)-*sec*-butyl)-5-oxo-4-(2-oxochroman-4-yl)-2,5-dihydro-1*H*-pyrrol-3-olate was designed. Molecular docking studies provided a docking score of −6.574 kcal/mol and an MM-GBSA value of −29.15 kcal/mol. Molecular dynamics simulation studies were carried out for 500 ns, providing better insights into the interaction. An RMSD change of 2.4 Å proved that there was a stable interaction and that there was no conformational change induced to the receptor. Metadynamics studies were performed to calculate the unbinding energy of the principal compound with NUDT5, which was found to be −75.171 kcal/mol. In vitro validation via a cytotoxicity assay (MTT assay) of the principal compound was carried out with quercetin as a positive control in the MCF7 cell line and with an IC_50_ value of 55.57 (+/−) 0.7 μg/mL. This work promoted the research of novel natural derivatives to discover their anticancer activity.

## 1. Introduction

The design of novel coumarin derivatives against breast cancer restricts ATP synthesis via the targeted NUDT5 antagonist. Breast cancer, a heterogeneous disease, is among the most frequently diagnosed diseases and is the second leading cause of death due to cancer among women after lung cancer. Breast cancer refers to cancers that develop in the breast tissue, most commonly in the inner lining of the milk ducts or in the lobules that supply milk to the ducts. It is the most common type of cancer in females [1]. Because of its significant impact on the population, this disease is a critical public health issue that necessitates additional molecular research to define its prognosis and specific treatment. Basic research is required to complete this task, and cell lines are used extensively in many aspects of laboratory research, particularly in in vitro models in cancer research [2]. Ninety percent of breast cancers are adenocarcinomas, which arise from the glandular tissue. Within this broad category, there is a great degree of variation. For instance, there are about 30 different subtypes of adenocarcinomas.

Phytoactives (plant-based derivatives) and their derivatives are safer than synthetic compounds in combating chemoresistance. Coumarin derivatives are natural compounds found in their free form or as a heteroside in plants. To date, 800 coumarin derivatives have been discovered in nature spread among 600 genera and 100 families. They are a type of naturally occurring plant metabolite with numerous biological functions. These coumarin derivatives have few side effects and are effective anticancer medications. In this regard, coumarin-derived medicines have shown promise as anticancer agents. Mechanistic studies have revealed that coumarins may cause self-programmed cancer cell death (apoptosis) through a variety of pathways, suggesting that coumarin-based derivatives might be used to treat a variety of malignancies, including drug-resistant and multidrug-resistant tumors [3]. These compounds have shown an incredible ability to modulate putative anticancer actions based on diverse substitution patterns [4]. Furthermore, because coumarin derivatives are abundant in nature, they are used mostly for the creation of new anticancer drugs, as they easily interact with a wide range of enzymes and receptors in cancer cells via weak bond interactions [5].

In the current work, a template-based design of the coumarin derivative was designed to target the ADP-sugar pyrophosphatase (NUDT5/NUDIX5) protein. NUDIX hydrolases are a group of nucleotide-metabolizing enzymes that play important roles in both health and sickness [6,7,8]. MTH1 (NUDT1, or NUDIX hydrolase 1), the best-studied NUDIX enzyme, is a nucleoside triphosphate pool sanitizer that protects nucleic acids’ integrity by decomposing oxidized purine nucleotides [9,10,11]. NUDT5 (NUDIX hydrolase 5, or NUDIX5), like MTH1, has been connected to the critical nucleotide metabolism and cancer pathways [12,13].

NUDT5′s primary substrates have been identified as 8-oxo-dGDP and adenosine 5′diphosphoribose (ADPR). While there is evidence that NUDT5 can hydrolyze 8-oxo-dGDP under basic conditions (pH 10), its physiological role in the 8-oxo-guanine metabolism has not been thoroughly investigated [14]. On the contrary, the hydrolysis of potential oxidized nucleotides and nucleotide-sugar substrates was carried out by MTH1 and NUDT5, as measured by the enzyme-coupled malachite green assay (MG assay), at a pH of 7.5 [15].

Along similar lines, the cloning and characterization studies of NUDT5 were performed at the optimal pH of 7 with the purified recombinant NUDT5 catalyzed hydrolysis of the two major substrates ADP-ribose and ADP-mannose having K_m_ values of 32 and 83 μm, respectively [16].

Recent research has revealed another function of NUDT5: it drives nuclear ATP synthesis, which may play a role in breast cancer. Previously, NUDT5 was discovered to catalyze the hydrolysis of 5′diphosphoribose (ADP-ribose, ADPR) into ribose-5-phosphate (R5P) and adenosine 5′-monophosphate (AMP) [15], demonstrating that, in the presence of pyrophosphate, NUDT5-catalyzed ADPR hydrolysis can generate both AMP and ATP. Nuclear ATP is the source of energy for fundamental biological processes such as chromatin remodeling and transcriptional change. Hormones such as estrogen and progestin can trigger such processes, which may be carcinogenic [17], and it was discovered that progestin- or estrogen-induced nuclear ATP increases, chromatin remodeling, and gene transcription changes in the breast cancer cell lines T47D and MCF7 are dependent on NUDT5 activity. NUDT5 has also been found to be overexpressed in breast cancer patients, and this is associated with a worse prognosis as well as a higher risk of recurrence and metastasis. These findings revealed that NUDT5 plays an important role in the estrogen signaling pathway and is, thus, involved in the pathogenesis of breast adenocarcinomas [18].

In the current work, a template-based approach was followed to design novel coumarin derivatives. The interactions between NUDT5 and coumarin were used to enumerate the novel derivatives, and anticancer activity was verified in the MCF-7 cell line. This work promoted the research of novel natural derivatives to discover their anticancer activity.

## 2. Results and Discussion

### 2.1. Protein Surface Analysis—Domain Analysis Provides Crucial Insights on Binding Site

**Figure 1:**
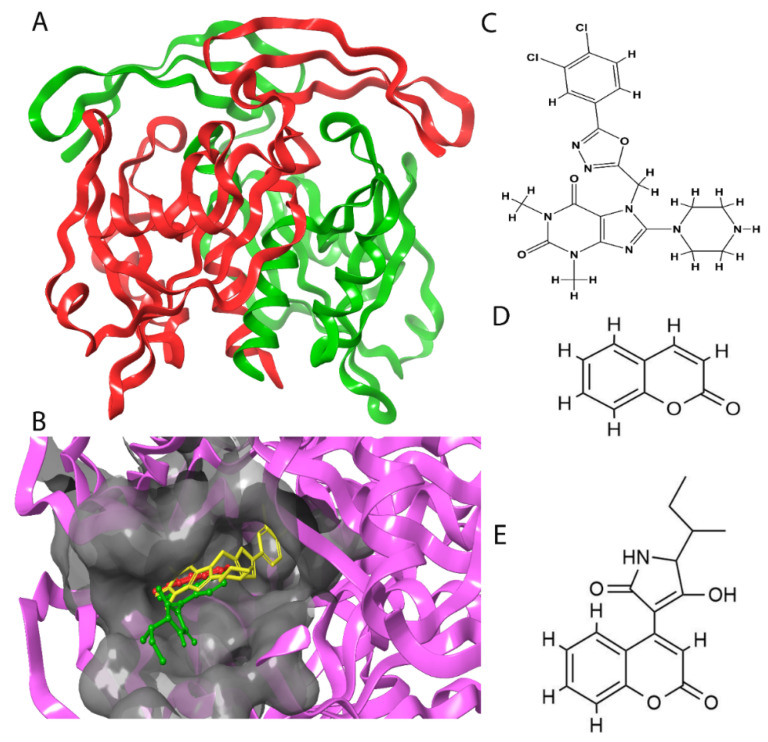
Structure visualization of receptors and compounds. (**A**) Three-dimensional structure of 5WNH with chain A highlighted in red and chain B highlighted in green. (**B**) Binding pose comparison of the binding mode comparison of TH5427 (green), coumarin (red), and principal compound (coumarin derivative, yellow) along with the protein (faded salmon). The binding pocket/surface is shown in gray. Both images A and B were obtained from Maestro v13.2. (**C**) Two-dimensional Fischer representation of TH5427. (**D**) Two-dimensional Fischer representation of coumarin. (**E**) Two-dimensional Fischer representation of coumarin derivative (principal compound).

The 5NWH shown in Figure 1A had a sum positive surface area of 8836.52 Å^2^ and a sum negative surface area of 6011.87 Å^2^. The amino acids in the domain are donors in nature and had a sum area of 2774.60 Å^2^ compared to a sum acceptor area of 3979.62 Å^2^. A total of 78 protein patches were analyzed using a Protein Surface Analyzer. With inputs from the ligand interaction diagram and the surface area, the surface was sorted into seven patches, i.e., 23, 76, 8, 4, 71, 45, and 31, based on the nine amino acids analyzed (Trp A:28, Trp B:46, Glu B:47, Glu A:166, Arg A:51, Arg A:84, Leu A:98, Ala A:96, and Glu A:116). It was observed that the ligand was spread across the positive, negative, and hydrophobic patches. The key interaction sites were mainly Trp A:28, Trp B:46, Glu B:47, and Arg A:51. With a higher accessibility and a surface area of 243.16 Å^2^, a positive patch of Trp A:28 and Arg A:51 had two arginine residues (Arg 44 and Arg 51), accounting for a total of 27% and 28% of the surface area, respectively, and one tryptophan residue (Trp 28) accounted for 32% of the surface area. A positive patch of Glu B:47 had a surface area of 116.73 Å^2^. It had two arginine residues (Arg 54 and Arg 44), accounting for a total of 10% and 32% of the surface area, respectively. The observations of the domain suggested that the ligand was spread out into the positive, negative, and hydrophobic patches concerning the main interactions involved in the ligand interaction diagram.

The functional part of the protein was further analyzed and studied after having understood the structural interactions between NUDT5 and TH5247. A 2D Fischer projection of TH5427 is shown in Figure 1C. Trp 28 of chain A (Trp A:28) had the feature of a substrate binding site [19]. Trp 46 of chain B (Trp B:46) and Glu 47 of chain B (Glu B:47) with ID positions of 46 and 47 were region types that had substrate binding sites with dimeric partners [20,21]. Similarly, Arg 51 of chain A (Arg A:51) with the ID position of 51 was a binding site type that had a substrate that was shared with a dimeric partner [19,22]. These were the amino acid binding sites that were potentially involved in docking.

### 2.2. Coumarin Derivatives Were Designed from Lead Optimization

**Figure 2:**
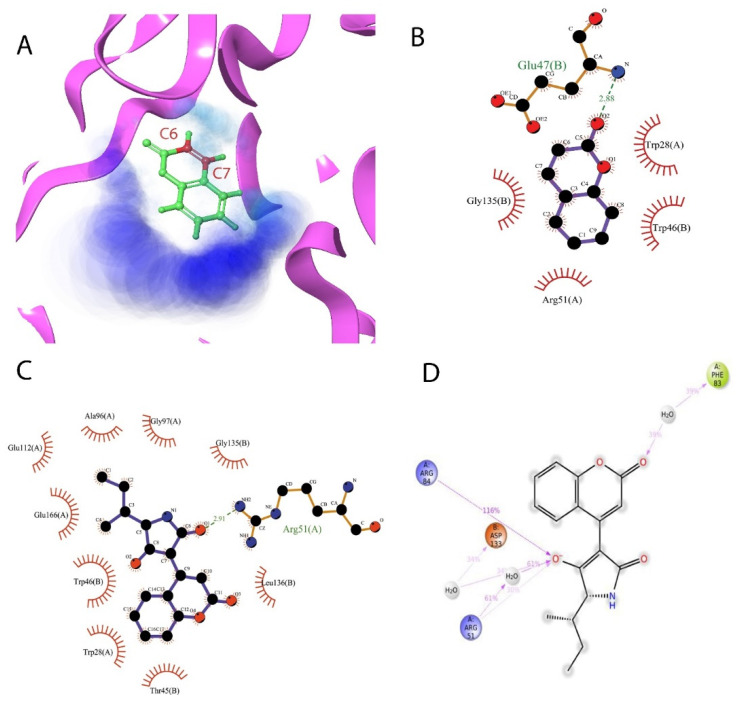
Enumeration and interaction profile of compounds with the receptor’s binding pocket. (**A**) The enumeration map of the binding pocket in the receptor is highlighted in magenta. Coumarin is colored in atom-specific colors: carbon in green, hydrogen in white, and oxygen in red. Dark blue is the site of solvent accessibility, and cyan is the site of the cavity. The atoms C6 and C7 are labeled in red and were utilized for enumeration. (**B**) Two-dimensional interaction profile of coumarin with residues in the binding site. (**C**) Two-dimensional interaction profile of principal compound with residues in the binding site. For both B and C, the hydrogen bond formed is shown in dashed green along with the bond length, and the hydrophobic interactions are highlighted in a shining red arch. (**D**) The 2D interaction profile of the principal compound shows the percentage of interaction with respective residues throughout the simulation period.

Coumarin was subjected to molecular docking to understand its binding interaction as a potential anticancer phytoactive. The 2D Fischer projection of coumarin is shown in Figure 1D. Two hydrogen bond interactions were observed between the coumarin-docked protein’s carbonyl oxygen atoms in Glu 47 (chain B) and Arg 51 (chain A). A stable hydrogen bond was visualized between the O2 of coumarin and the N1 of Glu47 with a bond length of 2.88 Å. It also had π–stacking interactions between Trp 28 (chain A) and Trp 46 (chain B), which aided in inhibitor binding. The 2D interaction profile is shown in Figure 2B.

Based on the interaction and the growth space available towards the C6 and C7 atoms of coumarin, enumeration was carried out. The cavity with a growth space is shown in Figure 2A. C7 was selected for the substitution of fragments. The closest amino acid was Arg 51, which is a donor in nature, and it was a potential site for hydrogen bond formation. Arg 51 was a part of the positive patch of the binding site, and, to achieve this, 148 R-group fragments were selected to form a hydrogen bond. The structures of the 148 fragments are provided in Supplementary Materials File S1.

The C7 site of coumarin with substitution with 148 R-groups yielded a total of 13 derivatives which exhibited a docking score similar to that of the native coumarin. The basic data can be found in Table 1, and the details, along with the structure, are available in Supplementary Materials File S2.

The derivative 0_34 showed an increase in the docking score of −6.574, which was better compared to that of the native coumarin, which had a score of −5.682. The derivative 0_34 was considered for further synthesis and, henceforth, is referred to as the principal compound. The IUPAC name of the principal compound is 5-(*sec*-butyl)-4-hydroxy-3-(2-oxo-2*H*-chromen-4-yl)-1*H*-pyrrol-2(5*H*)-one. The 2D Fischer projection of the coumarin derivative 0_34 (principal compound) is shown in Figure 1E.

The molecular interactions of NUDT5 with the principal compound were found to be composed of hydrogen bonds, π–π stacking, and π–cation interactions. The hydrogen bond interaction was found with Arg 51. It was observed that the O1 of the principal compound interacted with the NH2 of Arg 51 with an *H*-bond length of 2.91 Å. The π–π interaction was found with the Trp 46 of chain B, and the π–cation interaction was found with the Lys 27 of chain A. The 2D interaction profile is shown in Figure 2C.

The binding mode comparison of TH5427 (green), coumarin (red), and the principal compound (coumarin derivative, yellow) along with the protein (faded salmon) are highlighted in Figure 1B.

The structure–activity relationship (SAR) was a crucial factor in correlating the activity of TH5247 with the principal compound. Aligning both of the compounds to generate a common feature involved in the interaction profile suggested the presence of two acceptor oxygen atoms and two aromatic rings as a scaffold. As shown in Supplementary Materials File S3, the oxygen atom in the enumerated fragment was involved in the interaction with Arg 51, with a hydrogen bond length of 2.63 Å and 2.91 Å in TH5247 and the principal compound, respectively. The common residues in the binding pocket showing hydrophobic interactions were Ala 96 and Trp 28 in chain A along with Trp 46 and Thr 45 in chain B. The binding site alignment, with a threshold at 5 Å in the respective compounds, yielded an RMSD value of 0.00, suggesting the same binding pocket. 

### 2.3. Molecular Dynamics Simulations, MM-GBSA, and Metadynamics Studies Provided Insights into the Molecular Interactions and Stability of Binding and Unbinding

The complex of NUDT5 with the principal compound was subjected to molecular dynamics simulation studies for 500 ns. In the previously reported studies, it was reported that longer simulation runs with lower gradients have provided better insights into the binding stability and interaction profile of the complex [23,24].

For the simulation setup, a neutral environment was created with the addition of 21 Na^+^ ions with a total concentration of 34.720 mM. For the simulation run at 500 ns and at a gradient of 0.1 ns, a total of 10,000 frames were generated. 

**Figure 3:**
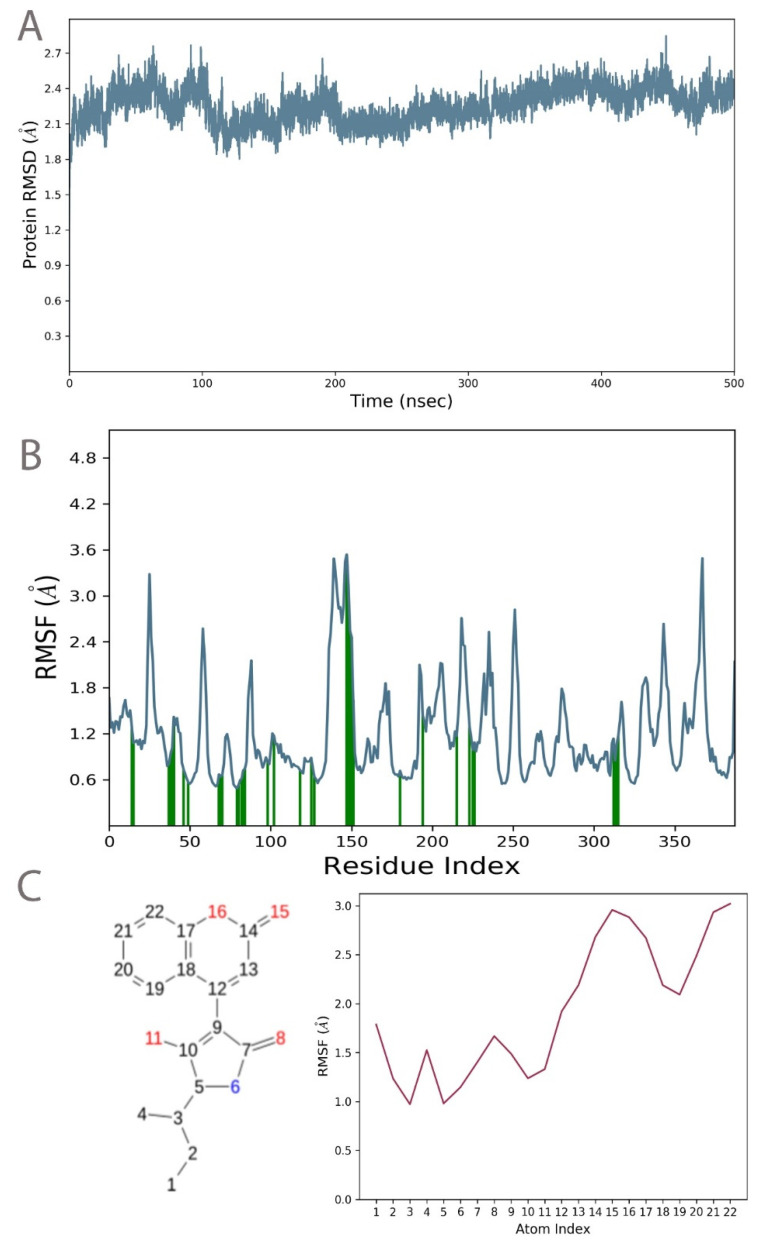
Plots depicting the results of molecular dynamics simulations. (**A**) Protein RMSD plot with the RMSD changes of C alpha atoms versus time in nanoseconds. (**B**) Protein RMSF plot of individual residues plotted against fluctuations observed. The ligand contacts are shown in green bars. (**C**) Ligand RMSF plot of the principal compound plotted with ligand atoms numbered in sequential order.

Protein root square mean deviation (RMSD) calculations provided insights into the stability of the protein and a confirmation of the interaction with the principal compound. A protein RMSD was calculated with the C alpha atoms of the backbone protein residues as a reference. A change of ~2.3 Å was observed throughout the simulation period with no fluctuations. The results suggested that the system was well equilibrated and that there were no conformational changes induced in the protein structure. The stable convergence of the RMSD values suggested the rigorous nature of the simulation period. The RMSD plot is available in Figure 3A.

Protein and ligand root mean square fluctuations (RMSF) equally provided crucial insights into the movement of the protein residues and the side atoms of the ligand. The protein RMSF suggested that no residues had fluctuation beyond 3.6 Å. Similarly, the ligand RMSF showed no movement of the ligand atoms beyond 3.0 Å. Both of these results emphasized the “tightness” of the ligand binding as well as no unexpected impacts on the protein conformation. The radius of gyration of 3.55 Å reaffirmed the stability of the ligand binding and the “fitness” within the binding pocket. The RMSF plot can be visualized, along with the interacting residues, in Figure 3B. The ligand RMSD and the corresponding atom numbers are present in Figure 3C.

The interactions of NUDT5 with the principal compound have been reported, and this interaction profile and its stability were reverified by plotting the protein–ligand contact plots using MD simulations. It could be observed that the principal compound had interactions throughout the simulation period with Arg 51 and Arg 84. Hydrogen bonds were observed with both residues with water-mediated interactions for a certain period. Ionic interactions were observed with Ala 96, Glu 112, and Glu 116. The 2D interaction profile of these interactions is shown in Figure 2D along with the percentage of time of the interaction periods. A detailed simulation report is provided in Supplementary Materials File S4.

The molecular mechanics–generalized Born surface area (MM-GBSA) provided a better understanding of the binding energy between the protein and the principal compound [25,26]. For MM-GBSA calculations, the simulation time frames were divided into 20 snapshots that were 500 frames apart. This provided optimal insight into the binding energy throughout the simulation period. The binding energy was found to be −29.15 (+/−4.86) kcal/mol. The MM-GBSA dg bind with no strain (NS) was found to be −31.42 (+/−4.59) kcal/mol. The individual values are tabulated in Table 2.

**Figure 4:**
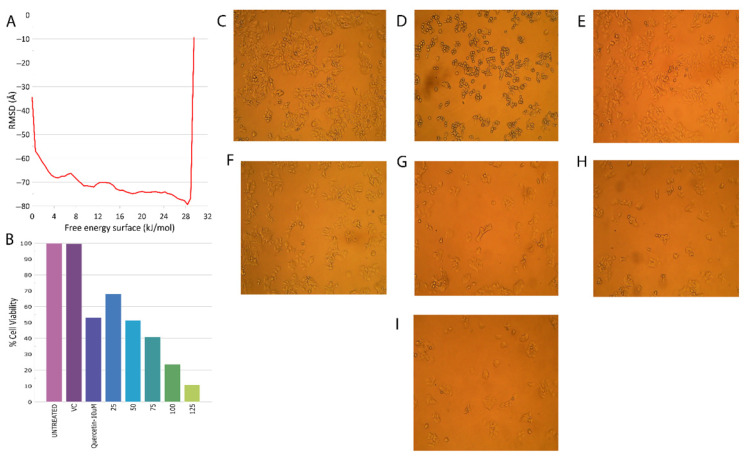
Cell viability was captured during the MTT assay. (**A**) The free energy surface values plotted against distance for metadynamics analysis. (**B**) The bar plot depicts the standard percentage of cell viability and that of samples at various concentrations. (**C**–**I**) Microscopic cell images of viability tests of untreated, positive control and of 25, 50, 75, 100, and 125 µg/mL concentrations, respectively.

Metadynamics studies were subjected to 50 ns. They were deployed for the calculation of the free energy surface [27,28]. This period has been observed in previous studies to be sufficient in calculating the unbinding energy. The calculation of the dissociation-free energy was performed using MATLAB. The calculated unbinding energy for the main compound with NUDT5 was −75.171 kcal/mol, which was higher compared to the binding energy found in the MM-GBSA studies. The free energy surface (FES) values were plotted against distance and can be visualized in Figure 4A.

### 2.4. MTT Assay

The in vitro validation via the cytotoxicity assay (MTT assay) of the principal compound 5NWH was carried out with quercetin at 10 µM as a positive control in the MCF7 cell line. The principal compound was dissolved using DMSO, and five dilutions of 25, 50.75, 100, and 125 µg/mL concentrations were prepared. The bar plot depicting the standard percentage of cell viability and that of the samples at various concentrations is shown in Figure 4B. The IC50 value of 55.57 (+/−) 0.7 μg/mL. The images of the cell viability tests are shown in Figure 4C–I for the untreated, positive control and for 25, 50, 75, 100, and 125 µg/mL concentrations, respectively. MTT assay suggested that the given compound was significantly cytotoxic in nature against MCF7, and further studies are to be conducted to determine the molecular mechanism behind the anticancer properties of the compound in human breast cancer cells. These results provided a greater validation of the potential phytoactive anticancer activity.

## 3. Materials and Methods

### 3.1. Protein Surface Analysis—Domain Analysis CDD

#### 3.1.1. Protein Preparation and Ligand Preparation

The crystal structure of human NUDT5 protein 5NWH was obtained from RCSB Protein Data Bank (PDB) [29]. 5NWH [30,31] is an ADP-sugar pyrophosphatase with two chains, A and B, with a sequence length of 209. The protein was bound to the ligand TH5427 [32].

The Protein Preparation Workflow tool [33] on Maestro Schrodinger 2022-2 [34] was used to fix issues with the protein and to maintain the pH of the environment on top of determining ligands, metals, and cosolvents. To determine residues and het atoms and to validate valency, the protein was preprocessed. During preprocessing, bond ordering was assigned, and hydrogens were replaced. Prime MM-GBSA was utilized to compensate for the lack of side chains. Epik [35] was used to construct het states with pH values of 7.4+/−2.0. During structural refining, PROPKA [36] was utilized to assign hydrogen bonds at neutral pH (7.0). Structure minimization for less than 0.30 Å was achieved using the OPLS3e [37] force field.

For the ligand, in the current work, we started with coumarin as a template to derive novel derivatives.

#### 3.1.2. Virtual Screening

A receptor grid was created at the site of the NUDT5 ATP binding site, wherein the coumarin compound was subjected to docking studies.

Glide [38,39] was used to dock the coumarin as the ATP binding site of NUDT5 using the Extra Precision (XP) [40] mode of docking. 

The docking analysis provided information on the interaction complementarity, geometric complementarity, docking score, correlation with experimental values, ligand interaction diagrams, etc. Here, the ligand, along with the binding pocket amino acids, was focused on, and the interactions between the ligand and the protein were analyzed. These included noncovalent bonds; pi interactions; and good, bad, and ugly contacts/clashes. The geometric complementarity checked if the ligands fit well into the binding pocket. The molecules with the best docking scores had the highest number of interactions with the protein, and this can be viewed in the results table. Additionally, the ligand interaction diagram provided a 2D view of the interactions between the ligand and the protein. The best binding pose was selected for lead optimization.

### 3.2. Lead Enumeration and Optimization

The results obtained from coumarin docking studies were used for lead optimization. The protein and the ligand with the best docking scores were merged. The newly merged complex was incorporated into the workspace. Under the Lead Optimization task, the Ligand Designer option was launched. Ligand Designer automatically generated a grid and showed the growth space. It also specified areas that could be modified to interact with the protein and the solvent-exposed regions. The conformity was based on Lipinski’s rule of 5. Here, protein residues were shown as pharmacophores. In the Enumeration Settings Panel, the ligand-designer form-bond option was chosen. With enumeration, all possible substitutions at that atom position in the ligand that could form an H-bond with the particular amino acid were generated. This also provided information on the substitution, protein residue, and basic ADME properties all in one place.

### 3.3. Molecular Dynamics Simulation

Desmond [41], a free academic user’s software, was used to perform the molecular dynamics simulation for a period of 500 ns. The interaction complex was subjected to protein preprocessing and H-bond assignment with parameters similar to those mentioned earlier. The simulation system was built utilizing the system builder. The solvent model selected was TIP3P [42], and boundary conditions were defined by the orthorhombic box with minimized volume encapsulating the complex. The force field applied was OPLS3e. The system was neutralized by adding Cl^−^ or Na^+^ ions based on the system’s total charge. The detailed methodology of MD simulation studies and MM-GBSA can be found in our previous publications [43,44,45,46].

### 3.4. Molecular Mechanics–Generalized Born Surface Area Calculations

The trajectory files from the MD simulations were used as the input for GBSA calculations [47]. The trajectory consisting of 500 ns generated 10,000 frames. The structures were extracted every 500 frames, and a total of 20 structures were subjected to GBSA energy calculations.

VSGB 2.0 solvation model [47] was used for calculations. MM-GBSA generated a lot of energy properties. These properties reported energies for the ligand, receptor, and complex structures as well as energy differences relating to strain and binding, and they were broken down into contributions from various terms in the energy expression.

There were five fundamental energy calculations done in Prime MM-GBSA: optimized free receptor (= “Receptor”), optimized free ligand (= “Ligand”), optimized complex (= “Complex”), receptor of minimized/optimized complex, ligand of minimized/optimized complex.

From these energies, MM-GBSA dG Bind = Complex − Receptor − Ligand, and MM-GBSA dG Bind (NS) = Complex − Receptor (from optimized complex) − Ligand (from optimized complex). MM-GBSA dG Bind − Rec Strain − Lig Strain values are of crucial importance [48].

### 3.5. Well-Tempered Metadynamics Study

The Metadynamics module of Desmond was used to carry out the analysis. The height and width of the Gaussian potential as well as the interval at which the Gaussians were added were the parameters that influenced the simulation’s accuracy. The height-to-interval ratio had a minor effect on accuracy; nonetheless, lesser values of this ratio boosted accuracy slightly. During a free MD run, the Gaussian’s breadth should be about 1/4 to 1/3 of the average fluctuations of the collective variable.

A wall with a CV is equal to the sum of the complex’s biggest dimensions. The wall was set to 30 in this investigation, which contained the entire receptor–ligand complex.

The time interval between injections of Gaussians was set to 0.09 picoseconds (ps). Temperature and pressure in the simulation were set to 310 K and 1.01325 bar, respectively. The simulation time was set to 50 ns.

The detailed protocol is available in [49]. Calculation of dissociation free energy (DFE) from the free energy surface (FES) data is available in [50].

### 3.6. Synthesis of 5-(sec-butyl)-4-hydroxy-3-(2-oxo-2H-chromen-4-yl)-1H-pyrrol-2(5H)-one

The detailed method for the synthesis of the title compound is available in [51] along with its characterization and DHODH inhibition. The structure is available in PubChem bearing CID 164182077 [52].

### 3.7. In Vitro Cytotoxicity Assay

MTT assay [53] was carried out on the MCF7 cell line (obtained from Stellixir Biotech Pvt. Ltd., Karnataka, India). Seed 200 μL cell suspensions in a 96-well plate were plated at the required cell density (20,000 cells per well) without the test agent and were incubated for about 24 h. Appropriate concentrations of the test agent were added, and the plate was incubated for 24 h at 37 °C in a 5% CO_2_ atmosphere. After the incubation period, the plates were taken out of the incubator, and the spent media was removed. MTT reagent was further added to a final concentration of 0.5 mg/mL of the total volume, and the plate was wrapped with aluminum foil to avoid exposure to light. 

The plates were kept in the incubator for an incubation period of 2 h. After incubation, the MTT reagent was removed, and then 100 μL of solubilization solution (DMSO) was added. Gentle stirring in a gyratory shaker was carried out, as it enhanced dissolution. Occasionally, pipetting up and down may be required to completely dissolve the MTT formazan crystals, especially in dense cultures. The absorbance was read using a spectrophotometer or an ELISA reader at 570 nm wavelength, and the IC50 value was determined using a linear regression equation, i.e., Y = Mx + C. Here, Y = 50, and M and C values were derived from the viability graph.

## 4. Discussion

The role of NUDT enzymes is well studied in breast cancer. Breast cancer stem cells (BCSCs) are known to promote the epithelial-to-mesenchymal transition (EMT), metastatic colonization, and proliferation. As a result, gaining a better knowledge of the proteins and signaling pathways involved in the metastatic process may lead to treatment prospects [54]. 

Coumarin has been used as a potential scaffold in designing novel derivatives as potential antibreast cancer compounds. Recently, coumarin derivatives have been used as MDM2 inhibitors [55]. The drug inhibited MDM2 and the antiapoptosis proteins Bcl-2 and Bcl-xL while increasing p53 and the proapoptosis protein BAX, inducing cell cycle arrest during the G2/M phase and activating Caspase-9 to cause apoptosis. 

In a similar work, human invasive breast ductal carcinoma cells were resistant to 18 coumarin derivatives. The work implied that tested coumarin compounds might inhibit tumor mass development [56].

The approved drugs were used to repurpose their activity against NUDT5. The IC_50_ values of nomifensine and isoconazole, which may interact with the important functional residues in NUDT5, were lower than those of the recognized antiestrogens raloxifene and tamoxifen. The foregoing findings demonstrated the utility of the medication repositioning strategy in the discovery of new NUDT5 inhibitors [57].

Our current work added to the scientific knowledge of the research community, as coumarin derivatives targeted the NUDT5 pathway of breast cancer cells.

## 5. Conclusions

Novel and retrosynthetic approaches have been showcased which promote the enumeration of novel coumarin derivatives [58,59]. 

Although most natural coumarins have significant restrictions in their usage due to their hepatotoxic impact, molecular modifications have produced comparatively safe analogs with a higher potency and, consequently, a superior therapeutic index. Significant favorable findings in the anticancer activity screening were obtained in coumarin structure–activity investigations with the addition of substituents at different locations of the coumarin core. As a result, developing novel anticancer compounds by adding suitable functional groups to various places around the coumarin core is an interesting topic of research.

In the current research work that was carried out, the major focus was on identifying novel coumarin derivatives as potential anticancer agents targeting the ATP synthesis pathway. Following the pharmacophore template-based approach, coumarin derivatives were designed for effective binding with NUDT5. The MM-GBSA and metadynamics studies that were carried out set a benchmark for future studies to follow the protocol to gain more insights into the binding and unbinding energies of the compounds. From the SAR analysis, the scope of enumeration at the O3 site in the principal compound (Supplementary Materials File S3) was used for better binding and inhibition of NUDT5. The work showed promising results and can be used as a template for better enumeration in the future.

## Figures and Tables

**Table 1:** List of enumerated coumarin derivatives along with their docking scores in kcal/mol.

Sl No.		Compound	Docking Score in kcal/mol
1	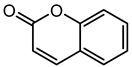	Coumarin	−5.682
2	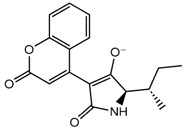	Derivative 0_34	−6.574
3	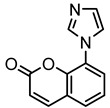	Derivative 0_10	−5.556
4	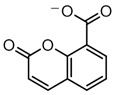	Derivative 0_15	−5.48
5	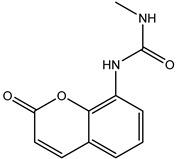	Derivative 0_29	−5.464
6	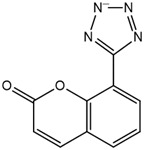	Derivative 0_16	−5.326
7	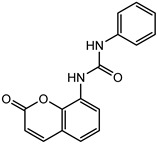	Derivative 0_30	−5.326
8	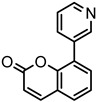	Derivative 0_7	−5.095
9	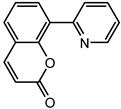	Derivative 0_6	−4.874
10	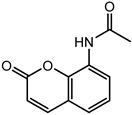	Derivative 0_24	−4.861
11	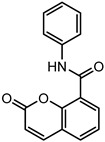	Derivative 0_26	−4.826
12	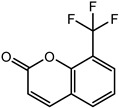	Derivative 0_3	−4.8
13	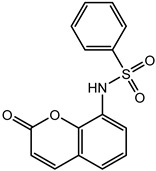	Derivative 0_28	−4.121

**Table 2:** GBSA binding scores with and without strain in kcal/mol for 20 snapshots at equal intervals of the simulation period.

Sl No.	Time (ns)	MM-GBSA (dG) Bind	MM-GBSA (dG) Bind (NS)
1	25	−33.67971994	−31.210146652
2	50	−29.43619808	−31.28268495
3	75	−31.57020652	−31.144059824
4	100	−31.73330412	−31.565264555
5	125	−37.21172526	−30.7391644435
6	150	−38.76721981	−30.6644028927
7	175	−23.44127048	−31.763695976
8	200	−22.18155238	−31.329428519
9	225	−28.85833401	−31.375644288
10	250	−32.05286903	−31.542408358
11	275	−19.49123229	−31.229535288
12	300	−32.10821288	−31.26827398
13	325	−28.05239437	−31.371629673
14	350	−31.738841	−31.51463875
15	375	−30.89106052	−31.568177393
16	400	−28.96861394	−31.604775712
17	425	−24.37966333	−31.537286241
18	450	−27.73930405	−30.7200561051
19	475	−21.82038688	−31.502836871
20	500	−28.89739628	−30.3340481743

## Data Availability

The protein structure used in the analysis is available in the PDB database with PDB ID 5WNH (http://doi.org/10.2210/pdb5WNH/pdb (accessed on 14 March 2022)). The structures for coumarin (CID: 323) and TH5427 (CID:132472992) are available in the respective PubChem CID. The principal compound has been uploaded in PubChem, and it is available with CID 164182077.

## Supplementary Materials

The following supporting information can be downloaded at: https://www.mdpi.com/article/10.3390/molecules28010089/s1, Supplementary Materials File S1: A tabular representation of 148 fragments used for the enumeration of coumarin. Supplementary Materials File S2: A tabular representation of native coumarin structure along with the stable in silico derivatives. Supplementary Materials File S3: Structure–activity comparison of TH5427 with principal compound. (**A**) Two-dimensional representation of TH5427 with common features highlighted in red. (**B**) Two-dimensional representation of principal compound with common features highlighted in red. (**C**) The interaction profile of TH5427 with NUDT5. (**D**) The interaction profile of the principal compound with NUDT5. For both C and D, the red semicircle suggests the hydrophobic residues. Supplementary Materials File S4: A detailed report of the MD simulation results.

## Sample Availability

Samples of the principal compound are available from the authors on request for a specified price.

## References

[1] Ganesh S., Vennila J.J., Scholz H. (2010). An Overview and Perspectives of Wilm’s Tumor. Int. J. Cancer Res..

[2] Raica M., Ceausu A.R., Cimpean A.M., Comşa Ş., Sarb S. (2021). Chloride Intracellular Channel Protein 1 (CLIC1), E-cadherin and P-cadherin Define Distinct Subclasses of HER2, Luminal B and Triple-negative Breast Cancer. Anticancer Res..

[3] Wang G., Sun S., Wu B., Liu J. (2021). Coumarins as Potential Anti-drug Resistant Cancer Agents: A Mini Review. Curr. Top. Med. Chem..

[4] Rawat A., Reddy A.V.B. (2022). Recent advances on anticancer activity of coumarin derivatives. Eur. J. Med. Chem. Rep..

[5] Song X., Fan J., Liu L., Liu X., Gao F. (2020). Coumarin derivatives with anticancer activities: An update. Arch. Der Pharm..

[6] Bessman M.J., Frick D.N., O’Handley S.F. (1996). The MutT Proteins or “Nudix” Hydrolases, are a Family of Versatile, Widely Distributed, “Housecleaning” Enzymes. J. Biol. Chem..

[7] McLennan A.G. (2005). The Nudix hydrolase superfamily. Cell. Mol. Life Sci. CMLS.

[8] Mildvan A., Xia Z., Azurmendi H., Saraswat V., Legler P., Massiah M., Gabelli S., Bianchet M., Kang L.-W., Amzel L. (2005). Structures and mechanisms of Nudix hydrolases. Arch. Biochem. Biophys..

[9] Mo J.Y., Maki H., Sekiguchi M. (1992). Hydrolytic elimination of a mutagenic nucleotide, 8-oxodGTP, by human 18-kilodalton protein: Sanitization of nucleotide pool. Proc. Natl. Acad. Sci. USA.

[10] Tsuzuki T., Egashira A., Igarashi H., Iwakuma T., Nakatsuru Y., Tominaga Y., Kawate H., Nakao K., Nakamura K., Ide F. (2001). Spontaneous tumorigenesis in mice defective in the *MTH1* gene encoding 8-oxo-dGTPase. Proc. Natl. Acad. Sci. USA.

[11] Anderson K., Iyidogan P. (2014). Faculty Opinions Recommendation of MTH1 Inhibition Eradicates Cancer by Preventing Sanitation of the dNTP Pool.

[12] Zhang L.-Q., Song X.-N., Dai D.-P., Zhou X.-Y., Gan W., Takagi Y., Hayakawa H., Sekiguchi M., Cai J.-P. (2013). Lowered Nudix type 5 expression leads to cellular senescence in IMR-90 fibroblast cells. Free Radic. Res..

[13] Zhang L.-Q., Dai D.-P., Gan W., Takagi Y., Hayakawa H., Sekiguchi M., Cai J.-P. (2011). Lowered Nudix type 5 (NUDT5) expression leads to cell cycle retardation in HeLa cells. Mol. Cell. Biochem..

[14] Helleday T. (2020). Abstract ES12-2: Targeted DNA repair in cancer and new NUDT5 inhibitors to block hormone signalling to target breast cancer. Cancer Res..

[15] Page B.D.G., Valerie N.C.K., Wright R.H.G., Wallner O., Isaksson R., Carter M., Rudd S.G., Loseva O., Jemth A.-S., Almlöf I. (2019). Author Correction: Targeted NUDT5 inhibitors block hormone signaling in breast cancer cells. Nat. Commun..

[16] Yang H., Slupska M.M., Wei Y.-F., Tai J.H., Luther W.M., Xia Y.-R., Shih D.M., Chiang J.-H., Baikalov C., Fitz-Gibbon S. (2000). Cloning and Characterization of a New Member of the Nudix Hydrolases from Human and Mouse. J. Biol. Chem..

[17] Wright R.H.G., Lioutas A., Le Dily F., Soronellas D., Pohl A., Bonet J., Nacht A.S., Samino S., Font-Mateu J., Vicent G.P. (2016). ADP-ribose–derived nuclear ATP synthesis by NUDIX5 is required for chromatin remodeling. Science.

[18] Le Dily F., Vidal E., Cuartero Y., Quilez J., Nacht A.S., Vicent G.P., Carbonell-Caballero J., Sharma P., Villanueva-Cañas J.L., Ferrari1 R. (2019). Hormone-control regions mediate steroid receptor-dependent genome organization. Genome Res..

[19] Zha M., Zhong C., Peng Y., Hu H., Ding J. (2006). Crystal Structures of Human NUDT5 Reveal Insights into the Structural Basis of the Substrate Specificity. J. Mol. Biol..

[20] Zha M., Guo Q., Zhang Y., Yu B., Ou Y., Zhong C., Ding J. (2008). Molecular Mechanism of ADP-Ribose Hydrolysis By Human NUDT5 From Structural and Kinetic Studies. J. Mol. Biol..

[21] Arimori T., Tamaoki H., Nakamura T., Kamiya H., Ikemizu S., Takagi Y., Ishibashi T., Harashima H., Sekiguchi M., Yamagata Y. (2011). Diverse substrate recognition and hydrolysis mechanisms of human NUDT5. Nucleic Acids Res..

[22] Pa V., Vijayaraghavareddy P., Uttarkar A., Dawane A., Sujitha D., Ashwin V., KC B., Niranjan V., Sheshshayee M.S., Anuradha M.C. (2022). Novel small molecules targeting bZIP23 TF improve stomatal conductance and photosynthesis under mild drought stress by regulating ABA. FEBS J..

[23] Patagar D., Uttarkar A., Patra S.M., Patil J.H., Kusanur R., Niranjan V., Kumar H.G.A. (2021). Spiro Benzodiazepine Substituted Fluorocoumarins as Potent Anti-Anxiety Agents. Russ. J. Bioorg. Chem..

[24] Uttarkar A., Kishore A.P., Srinivas S.M., Rangappa S., Kusanur R., Niranjan V. (2022). Coumarin derivative as a potent drug candidate against triple negative breast cancer targeting the frizzled receptor of wingless-related integration site signaling pathway. J. Biomol. Struct. Dyn..

[25] (2022). Akshay Uttarkar & Vidya Niranjan (2022) Brefeldin A variant via combinatorial screening acts as an effective antagonist inducing structural modification in EPAC2. Mol. Simul..

[26] Niranjan V., Uttarkar A., Murali K., Niranjan S., Gopal J., Kumar J. (2022). Mycobacterium Time-Series Genome Analysis Identifies AAC2′ as a Potential Drug Target with Naloxone Showing Potential Bait Drug Synergism. Molecules.

[27] Bussi G., Laio A., Tiwary P. (2018). Metadynamics: A Unified Framework for Accelerating Rare Events and Sampling Thermodynamics and Kinetics. Handbook of Materials Modeling.

[28] Pickup K.E., Pardow F., Carbonell-Caballero J., Lioutas A., Villanueva-Cañas J.L., Wright R.H.G., Beato M. (2019). Expression of Oncogenic Drivers in 3D Cell Culture Depends on Nuclear ATP Synthesis by NUDT5. Cancers.

[29] Berman H.M., Westbrook J., Feng Z., Gilliland G., Bhat T.N., Weissig H., Shindyalov I.N., Bourne P.E. (2000). The Protein Data Bank. Nucleic Acids Res..

[30] Carter M., Stenmark P. (2018). Potent Inhibitors of NUDT5 Silence Hormone Signaling in Breast Cancer.

[31] National Center for Biotechnology Information (2022). PubChem Compound Summary for CID 132472992. https://pubchem.ncbi.nlm.nih.gov/compound/132472992.

[32] Madhavi Sastry G., Adzhigirey M., Day T., Annabhimoju R., Sherman W. (2013). Protein and ligand preparation: Parameters, protocols, and influence on virtual screening enrichments. J. Comput.-Aided Mol. Des..

[33] (2021). Schrödinger Release 2022-2: Maestro, Schrödinger, LLC, New York, NY. https://www.schrodinger.com/products/maestro.

[34] Shelley J.C., Cholleti A., Frye L.L., Greenwood J.R., Timlin M.R., Uchimaya M. (2007). Epik: A software program for pK a prediction and protonation state generation for drug-like molecules. J. Comput. Mol. Des..

[35] Olsson M.H.M., Søndergaard C.R., Rostkowski M., Jensen J.H. (2011). PROPKA3: Consistent Treatment of Internal and Surface Residues in Empirical p*K*_a_ Predictions. J. Chem. Theory Comput..

[36] Harder E., Damm W., Maple J., Wu C., Reboul M., Xiang J.Y., Wang L., Lupyan D., Dahlgren M.K., Knight J.L. (2015). OPLS3: A Force Field Providing Broad Coverage of Drug-like Small Molecules and Proteins. J. Chem. Theory Comput..

[37] Friesner R.A., Banks J.L., Murphy R.B., Halgren T.A., Klicic J.J., Mainz D.T., Repasky M.P., Knoll E.H., Shelley M., Perry J.K. (2004). Glide: A New Approach for Rapid, Accurate Docking and Scoring. Method and Assessment of Docking Accuracy. J. Med. Chem..

[38] Halgren T.A., Murphy R.B., Friesner R.A., Beard H.S., Frye L.L., Pollard W.T., Banks J.L. (2004). Glide: A New Approach for Rapid, Accurate Docking and Scoring. Enrichment Factors in Database Screening. J. Med. Chem..

[39] Friesner R.A., Murphy R.B., Repasky M.P., Frye L.L., Greenwood J.R., Halgren T.A., Sanschagrin P.C., Mainz D.T. (2006). Extra Precision Glide: Docking and Scoring Incorporating a Model of Hydrophobic Enclosure for Protein−Ligand Complexes. J. Med. Chem..

[40] Bowers K.J., Chow E., Xu H., Dror R.O., Eastwood M.P., Gregersen B.A., Klepeis J.L., Kolossvary I., Moraes M.A., Sac-erdoti F.D. (2006). Molecular Dynamics-Scalable algorithms for molecular dynamics simulations on commodity clusters. Proceedings of the 2006 ACM/IEEE Conference on Supercomputing; Association for Computing Machinery.

[41] Mark P., Nilsson L. (2001). Structure and Dynamics of the TIP3P, SPC, and SPC/E Water Models at 298 K. J. Phys. Chem. A.

[42] Khangwal I., Skariyachan S., Uttarkar A., Muddebihalkar A.G., Niranjan V., Shukla P. (2021). Understanding the Xylooligosaccharides Utilization Mechanism of Lactobacillus brevis and Bifidobacterium adolescentis: Proteins Involved and Their Conformational Stabilities for Effectual Binding. Mol. Biotechnol..

[43] Skariyachan S., Ravishankar R., Gopal D., Muddebihalkar A.G., Uttarkar A., Praveen P.K.U., Niranjan V. (2020). Response regulator GacA and transcriptional activator RhlR proteins involved in biofilm formation of Pseudomonas aeruginosa are prospective targets for natural lead molecules: Computational modelling, molecular docking and dynamic simulation studies. Infect. Genet. Evol..

[44] Gopal D., Muddebihalkar A.G., Skariyachan S., Kaveramma P., Praveen U., Shankar R.R., Venkatesan T., Niranjan V. (2019). Mitogen activated protein kinase-1 and cell division control protein-42 are putative targets for the binding of novel natural lead molecules: A therapeutic intervention against *Candida albicans*. J. Biomol. Struct. Dyn..

[45] Skariyachan S., Muddebihalkar A.G., Badrinath V., Umashankar B., Eram D., Uttarkar A., Niranjan V. (2020). Natural epiestriol-16 act as potential lead molecule against prospective molecular targets of multidrug resistant Acinetobacter baumannii-Insight from in silico modelling and in vitro investigations. Infect. Genet. Evol..

[46] Ylilauri M., Pentikäinen O.T. (2013). MM-GBSA As a Tool To Understand the Binding Affinities of Filamin–Peptide Interactions. J. Chem. Inf. Model..

[47] Li J., Abel R., Zhu K., Cao Y., Zhao S., Friesner R.A. (2011). The VSGB 2.0 model: A next generation energy model for high resolution protein structure modeling. Proteins Struct. Funct. Bioinform..

[48] Ahmad S. (2022). Molecular dynamics simulation and docking analysis of NF-κB protein binding with sulindac acid. Bioinformation.

[49] Niranjan V., Uttarkar A. (2022). Well-tempered Metadynamics Protocol.

[50] Wang J., Ishchenko A., Zhang W., Razavi A., Langley D. (2022). A highly accurate metadynamics-based Dissociation Free Energy method to calculate protein–protein and protein–ligand binding potencies. Sci. Rep..

[51] PubChem [Internet] PubChem [Internet]. Bethesda (MD): National Library of Medicine (US), National Center for Biotechnology Information; 2004 PubChem Substance Record for SID 468535216, SID 468535216, Source: Vidya Lab, Department of Biotechnology, RV College of Engineering, Bengaluru. https://pubchem.ncbi.nlm.nih.gov/substance/468535216.

[52] Patagar D., Kusanur R., Sitwala N.D., Ghate M.D., Saravanakumar S., Nembenna S., Gediya P.A. (2019). Synthesis of Novel 4-Substituted Coumarins, Docking Studies, and DHODH Inhibitory Activity. J. Heterocycl. Chem..

[53] van Meerloo J., Kaspers G.J.L., Cloos J. (2011). Cell Sensitivity Assays: The MTT Assay. Methods in Molecular Biology.

[54] Wright R., Beato M. (2021). Role of the NUDT Enzymes in Breast Cancer. Int. J. Mol. Sci..

[55] Ahmed E.Y., Latif N.A.A., Nasr T., Awad H.M., Abdelhafez O.M. (2022). Design, synthesis, and molecular modeling of coumarin derivatives as MDM2 inhibitors targeting breast cancer. Chem. Biol. Drug Des..

[56] Gkionis L., Kavetsou E., Kalospyros A., Manousakis D., Sanz M.G., Butterworth S., Detsi A., Tirella A. (2021). Investigation of the cytotoxicity of bioinspired coumarin analogues towards human breast cancer cells. Mol. Divers..

[57] Tong X.-Y., Liao X., Gao M., Lv B.-M., Chen X.-H., Chu X.-Y., Zhang Q.-Y., Zhang H.-Y. (2020). Identification of NUDT5 Inhibitors From Approved Drugs. Front. Mol. Biosci..

[58] Thakur A., Singla R., Jaitak V. (2015). Coumarins as anticancer agents: A review on synthetic strategies, mechanism of action and SAR studies. Eur. J. Med. Chem..

[59] Patil S.N., Sanningannavar F., Navati B., Patil N., Kusanur R., Melavanki R. (2014). Photophysical characteristics of two novel coumarin derivatives: Experimental and theoretical estimation of dipole moments using the solvatochromic shift method. Can. J. Phys..

