# Supplementary material for: Design of Novel Coumarin Derivatives as NUDT5 Antagonists That Act by Restricting ATP Synthesis in Breast Cancer Cells
Source: Molecules. 2022 Dec 22;28(1):89. doi: 10.3390/molecules28010089 (PMC9822328; doi:10.3390/molecules28010089)
Supplement: Supplementary file 1 [file molecules-28-00089-s001.zip › Supplementary Materials File S1.pdf]

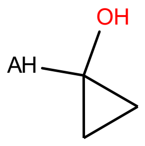

title Fragment 1

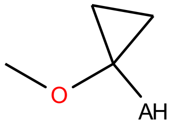

title Fragment 2

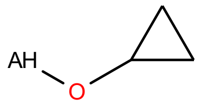

title Fragment 3

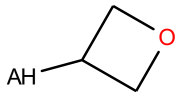

title Fragment 4

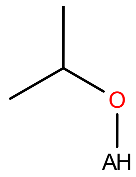

title Fragment 5

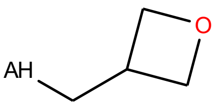

title Fragment 6

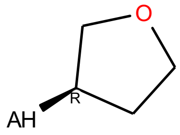

title Fragment 7

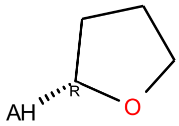

title Fragment 8

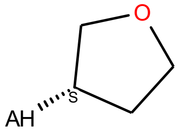

title Fragment 9

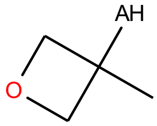

title Fragment 10

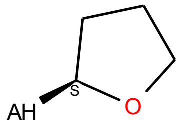

title Fragment 11

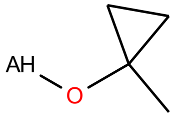

title Fragment 12

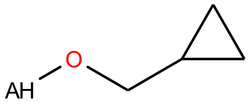

title Fragment 13

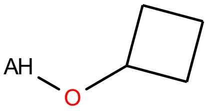

title Fragment 14

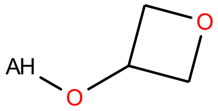

title Fragment 15

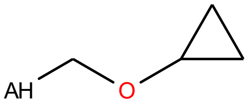

title Fragment 16

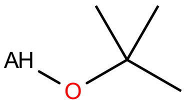

title Fragment 17

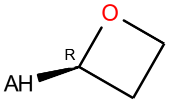

title Fragment 18

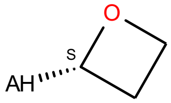

title Fragment 19

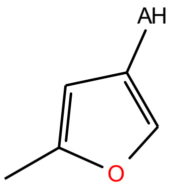

title Fragment 20

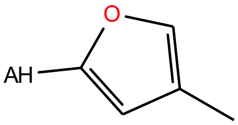

title Fragment 21

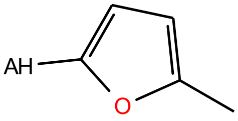

title Fragment 22

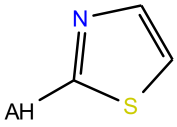

title Fragment 23

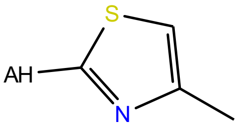

title Fragment 24

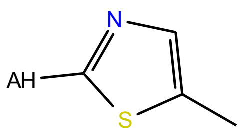

title Fragment 25

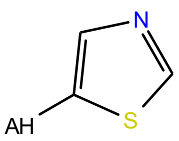

title Fragment 26

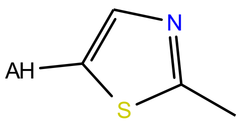

title Fragment 27

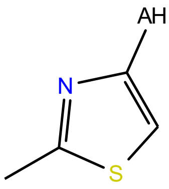

title Fragment 28

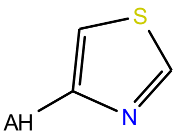

title Fragment 29

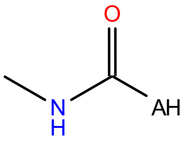

title Fragment 30

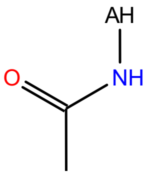

title Fragment 31

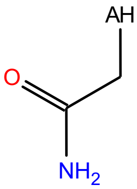

title Fragment 32

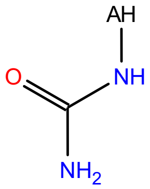

title Fragment 33

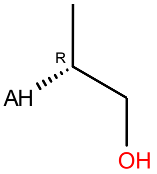

title Fragment 34

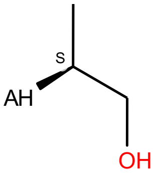

title Fragment 35

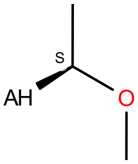

title Fragment 36

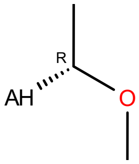

title Fragment 37

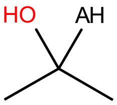

title Fragment 38

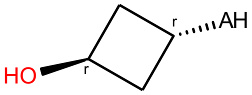

title Fragment 39

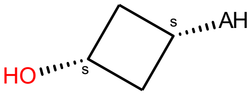

title Fragment 40

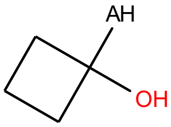

title Fragment 41

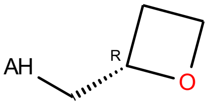

title Fragment 42

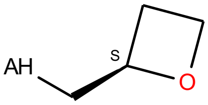

title Fragment 43

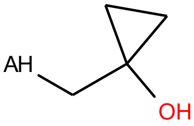

title Fragment 44

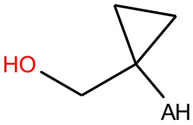

title Fragment 45

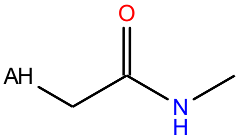

title Fragment 46

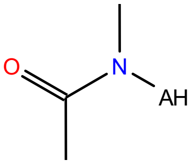

title Fragment 47

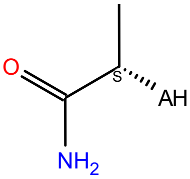

title Fragment 48

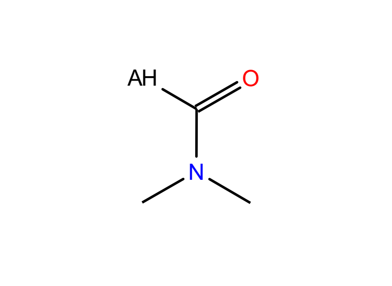

title Fragment 49

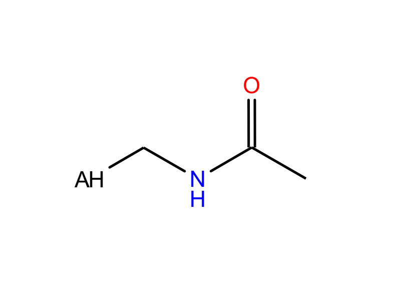

title Fragment 50

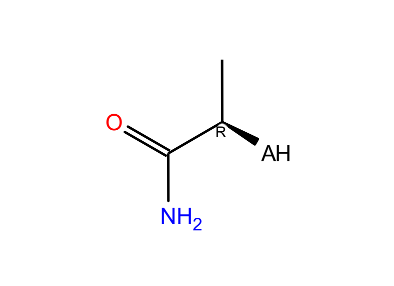

title Fragment 51

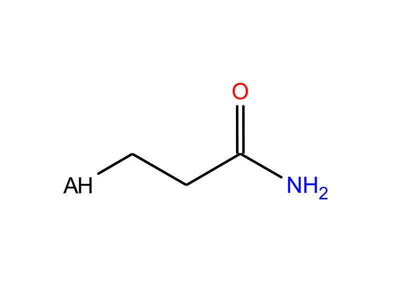

title Fragment 52

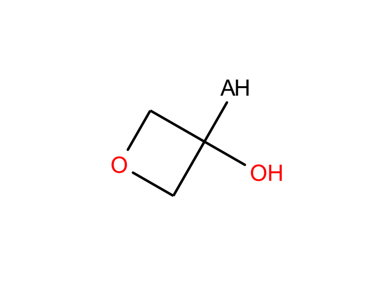

title Fragment 53

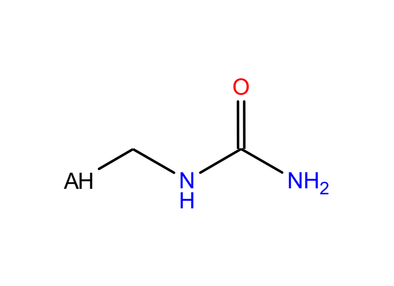

title Fragment 54

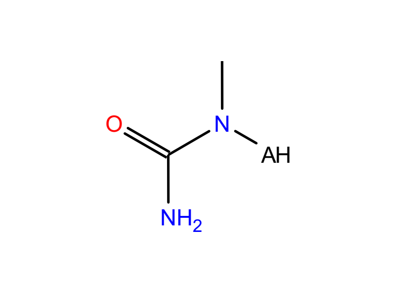

title Fragment 55

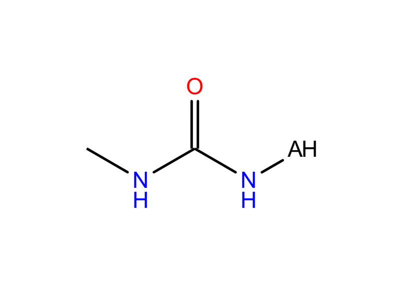

title Fragment 56

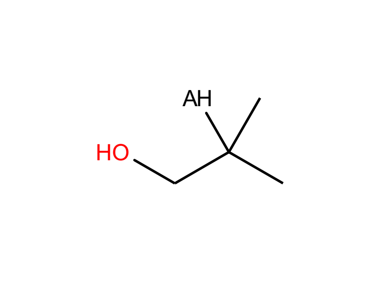

title Fragment 57

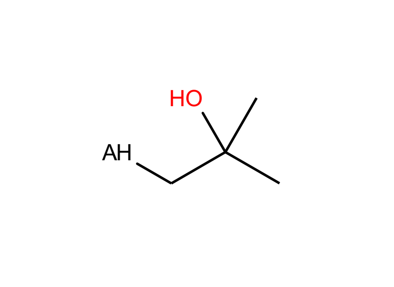

title Fragment 58

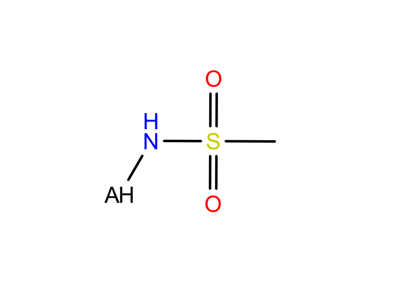

title Fragment 59

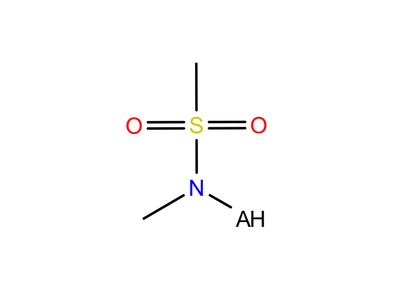

title Fragment 60

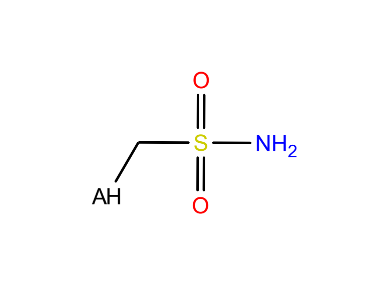

title Fragment 61

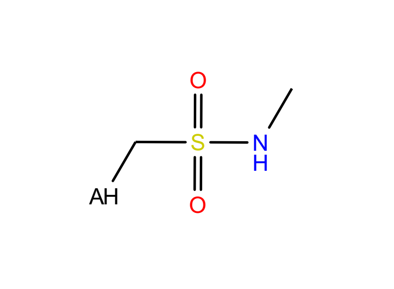

title Fragment 62

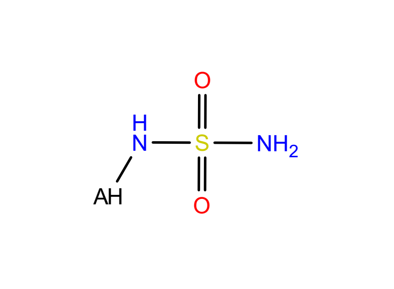

title Fragment 63

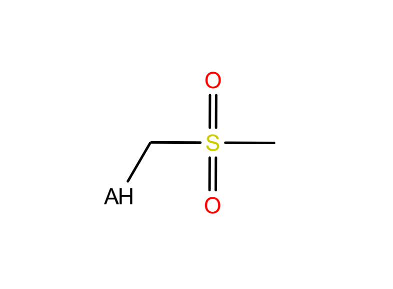

title Fragment 64

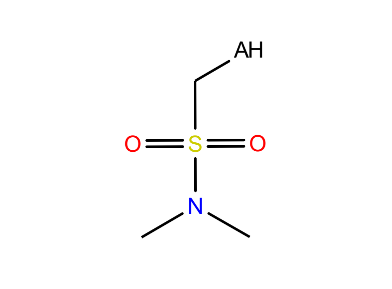

title Fragment 65

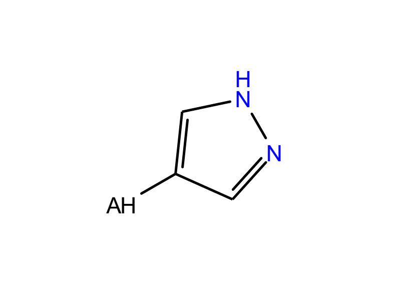

title Fragment 66

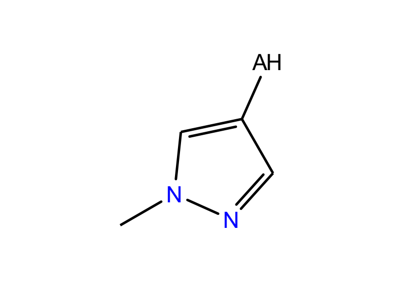

title Fragment 67

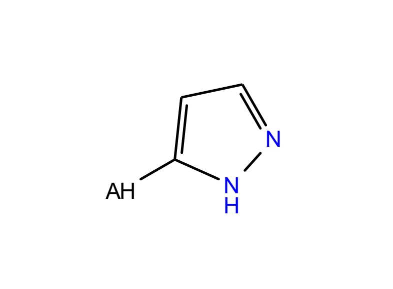

title Fragment 68

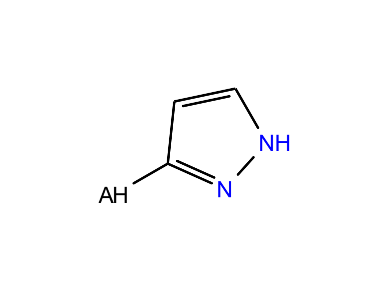

title Fragment 69

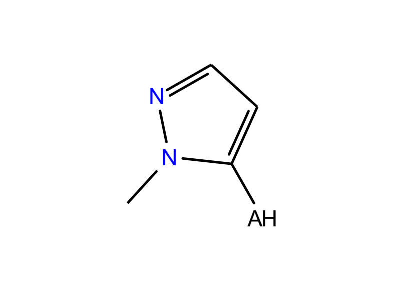

title Fragment 70

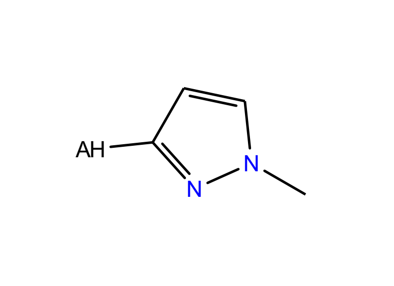

title Fragment 71

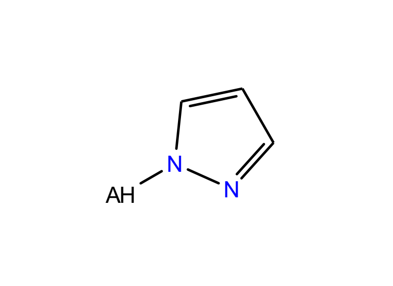

title Fragment 72

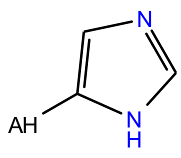

title Fragment 73

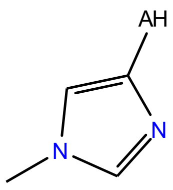

title Fragment 74

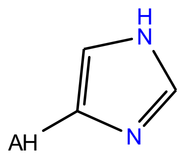

title Fragment 75

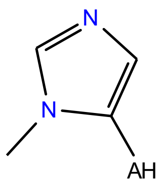

title Fragment 76

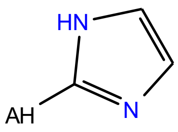

title Fragment 77

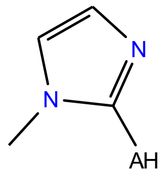

title Fragment 78

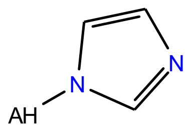

title Fragment 79

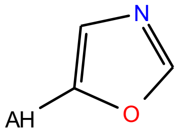

title Fragment 80

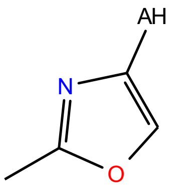

title Fragment 81

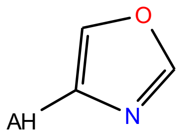

title Fragment 82

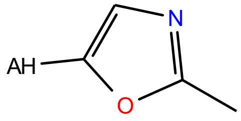

title Fragment 83

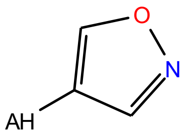

title Fragment 84

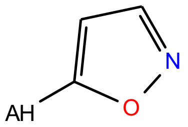

title Fragment 85

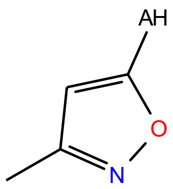

title Fragment 86

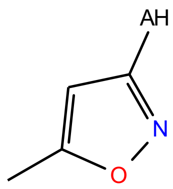

title Fragment 87

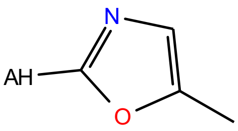

title Fragment 88

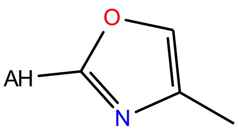

title Fragment 89

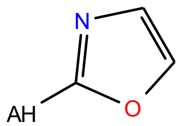

title Fragment 90

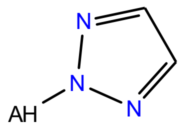

title Fragment 91

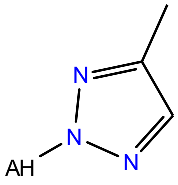

title Fragment 92

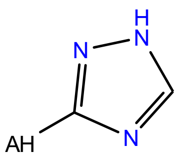

title Fragment 93

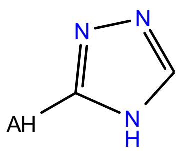

title Fragment 94

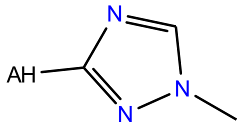

title Fragment 95

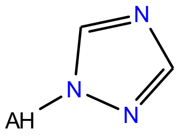

title Fragment 96

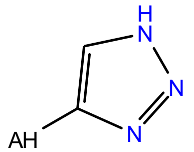

title Fragment 97

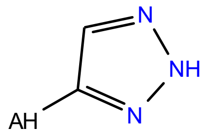

title Fragment 98

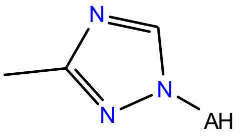

title Fragment 99

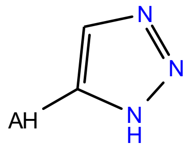

title Fragment 100

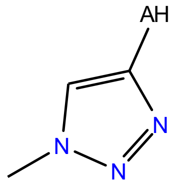

title Fragment 101

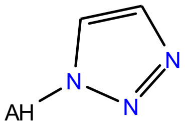

title Fragment 102

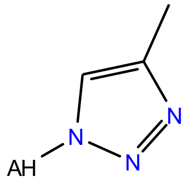

title Fragment 103

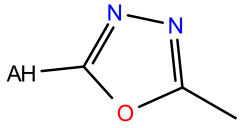

title Fragment 104

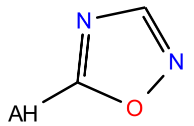

title Fragment 105

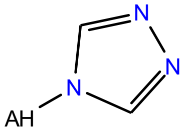

title Fragment 106

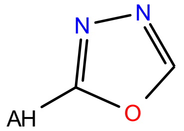

title Fragment 107

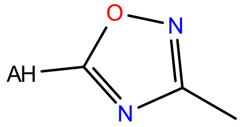

title Fragment 108

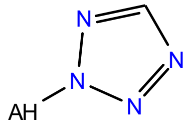

title Fragment 109

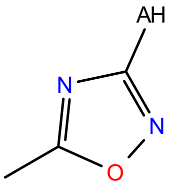

title Fragment 110

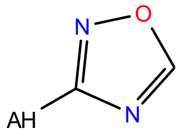

title Fragment 111

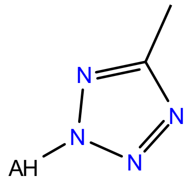

title Fragment 112

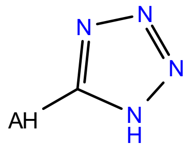

title Fragment 113

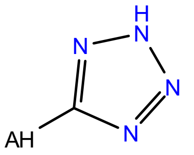

title Fragment 114

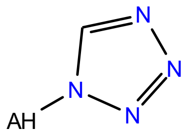

title Fragment 115

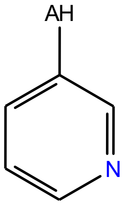

title Fragment 116

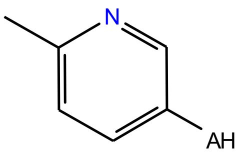

title Fragment 117

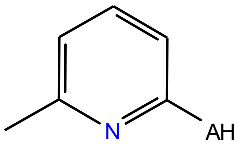

title Fragment 118

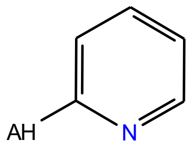

title Fragment 119

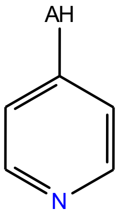

title Fragment 120

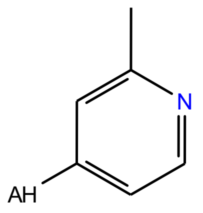

title Fragment 121

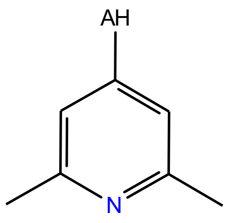

title Fragment 122

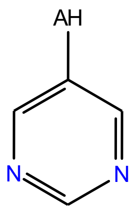

title Fragment 123

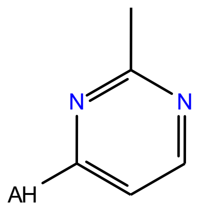

title Fragment 124

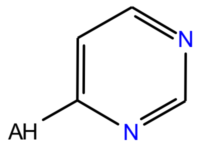

title Fragment 125

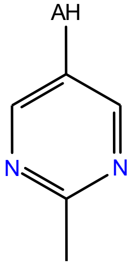

title Fragment 126

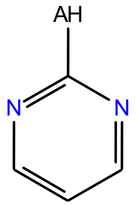

title Fragment 127

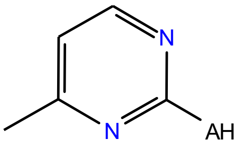

title Fragment 128

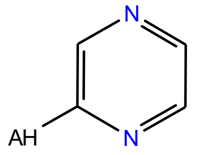

title Fragment 129

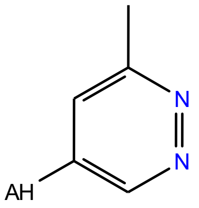

title Fragment 130

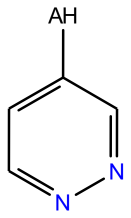

title Fragment 131

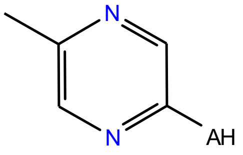

title Fragment 132

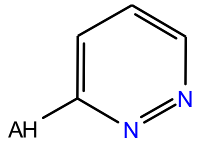

title Fragment 133

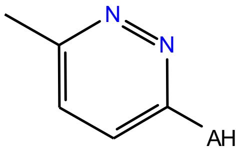

title Fragment 134

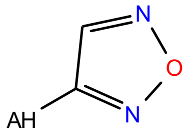

title Fragment 135

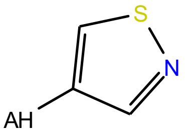

title Fragment 136

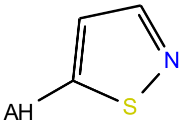

title Fragment 137

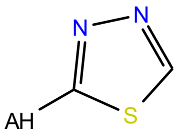

title Fragment 138

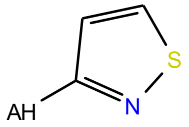

title Fragment 139

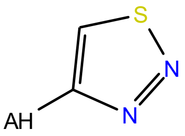

title Fragment 140

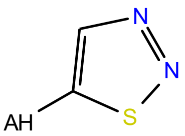

title Fragment 141

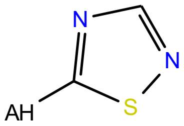

title Fragment 142

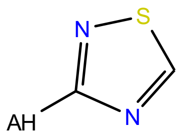

title Fragment 143

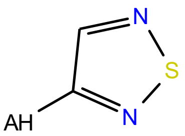

title Fragment 144

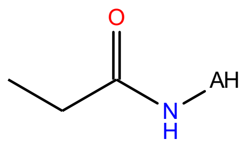

title Fragment 145

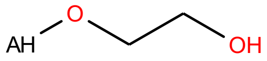

title Fragment 146

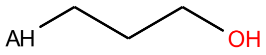

title Fragment 147

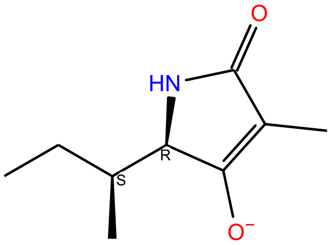

title Fragment 148
