# Supplementary material for: Design of Novel Coumarin Derivatives as NUDT5 Antagonists That Act by Restricting ATP Synthesis in Breast Cancer Cells
Source: Molecules. 2022 Dec 22;28(1):89. doi: 10.3390/molecules28010089 (PMC9822328; doi:10.3390/molecules28010089)
Supplement: Supplementary file 1 [file molecules-28-00089-s001.zip › Supplementary Materials File S2.pdf]

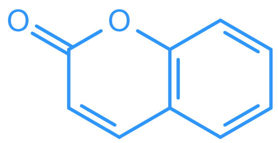

title	: Coumarin
docking score	-5.682

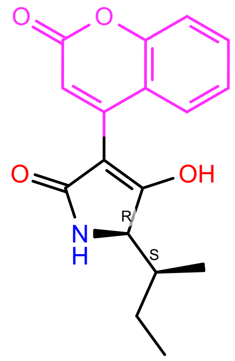

title	Dervivative 0_34
docking score	-6.574

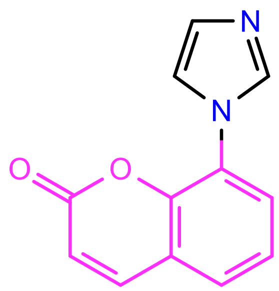

title	: Dervivative 0_10
docking score	-5.556

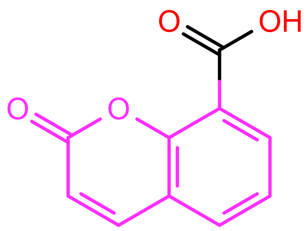

title	: Dervivative 0_15
docking score	-5.48

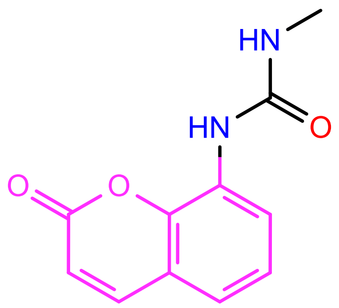

title	: Dervivative 0_29
docking score	-5.464

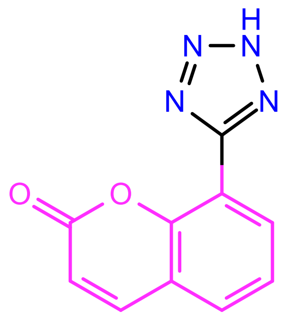

title	: Dervivative 0_16
docking score	-5.326

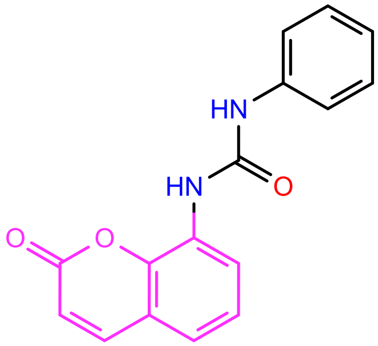

title	: Dervivative 0_30
docking score	-5.326

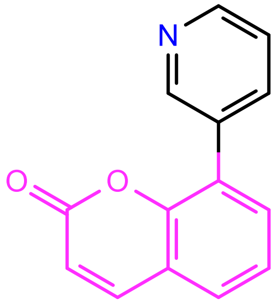

title	: Dervivative 0_7
docking score	-5.095

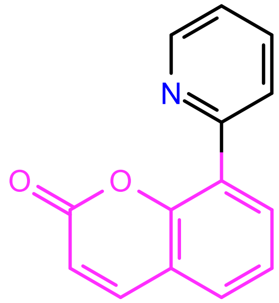

title	: Dervivative 0_6
docking score	-4.874

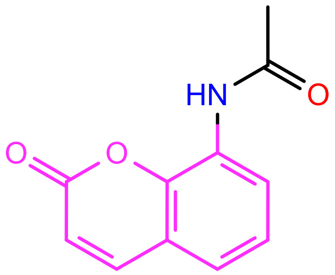

title	: Dervivative 0_24
docking score	-4.861

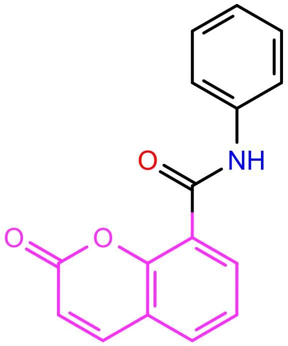

title	: Dervivative 0_26
docking score	-4.826

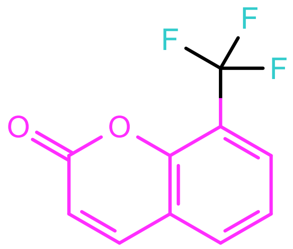

title	: Dervivative 0_3
docking score	-4.8

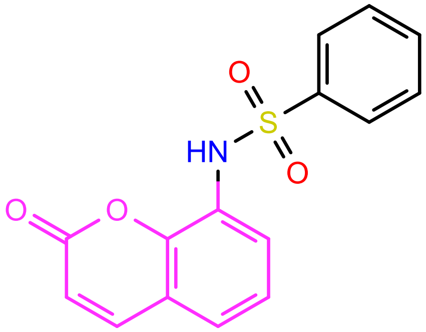

title : Dervivative 0_28		
docking score -4.121		
